# A randomized pragmatic feasibility trial to promote student perspective-taking on client physical activity level: a collaborative project

**DOI:** 10.1186/s40814-024-01547-8

**Published:** 2024-09-28

**Authors:** Lisa B. Hoplock, Michelle M. Lobchuk, Shaelyn M. Strachan, Gayle Halas, Cheryl Olfert, Sandra Webber, Joanne L. Parsons

**Affiliations:** 1 College of Nursing, University of Manitoba, Winnipeg, MB R3T 2N2, Canada; 2 Faculty of Kinesiology and Recreation Management, University of Manitoba, Winnipeg, MB R3T 2N2, Canada; 3 Faculty of Health Sciences, University of Manitoba, Winnipeg, MB R3E 0W3, Canada; 4 College of Rehabilitation Sciences, University of Manitoba, Winnipeg, MB R3E 0T6, Canada

**Keywords:** Perspective-taking, Empathy, Education, Feasibility

## Abstract

**Background:**

Health-care practitioners have opportunities to talk with clients about unhealthy behaviors. How practitioners approach these conversations involves skill to be effective. Thus, teaching health-care students to communicate empathetically with clients should promote effective client-practitioner conversations about health behavior change. The primary objective of this pilot trial was to assess the feasibility, acceptability, and appropriateness of a theoretically informed intervention designed to improve perspective-taking.

**Methods:**

For inclusion in this randomized mixed-methods parallel two-arm trial, participants needed to be a student at the investigators’ Canadian university and have completed course content on behavior change communication. Using a 1:1 allocation ratio, participants in Respiratory, Physical, and Occupational Therapy; Nurse Practitioner; and Kinesiology programs were randomly assigned to full or partial intervention conditions. Full intervention participants completed a perspective-taking workshop and practiced perspective-taking prior to an in-lab dialogue with a client-actor (masked to condition) about physical activity. Partial intervention participants received the workshop after the dialogue. We assessed feasibility and appropriateness by comparing recruitment rates, protocol, and psychometric outcomes to criteria. We assessed acceptability (secondary outcome) by analyzing exit interviews.

**Results:**

We screened and randomized 163 participants (82 = full intervention; 81 = partial intervention). We fell slightly short of our recruitment success criteria (10–15 participants per program) when 2/50 Occupational Therapy students participated. We met some but not all of our protocol criteria: Some full intervention participants did not practice perspective-taking before the dialogue, because they did not see anyone during the practice period or did not have a practice opportunity. Psychometric outcomes met the criteria, except for one measure that demonstrated ceiling effects and low reliability (Cronbach’s alpha < .70). There were no adverse events related to participation.

**Conclusions:**

The intervention should be largely feasible, appropriate, and acceptable to deliver. We suggest changes that are large enough to warrant conducting another pilot study. We outline recommended improvements that are applicable to researchers and educators interested in recruitment, adherence to home practice, and online uptake of the intervention.

**Trial registration:**

This trial was registered retrospectively on November 8, 2023, at https://clinicaltrials.gov/study/NCT06123507.

**Supplementary Information:**

The online version contains supplementary material available at 10.1186/s40814-024-01547-8.

## Key messages regarding feasibility

- What uncertainties existed regarding the feasibility?

We had uncertainties about the number of interested participants, and about protocol adherence and acceptability. We were also uncertain about the feasibility of completing the following on time: employee training, participant scheduling, and lab session protocols.

- What are the key feasibility findings?

We fell slightly short of our recruitment goal for Respiratory Therapy, Nurse Practitioner, and Occupational Therapy programs. Participants found the protocol acceptable and valued practicing in a welcoming in-lab environment. However, some in the full intervention condition had difficulty finding time and people to practice with. It was feasible to complete the protocol within the allotted lab time and to schedule sessions 1–2 weeks after recruitment. Participants in the full intervention condition may benefit from increased time between recruitment and scheduling to allow for more practice. The allotted time for training employees was feasible.

- What are the implications of the feasibility findings for the design of the main study?

The findings of this study provide important information about recruitment and adherence for a perspective-taking intervention for health-care students. In our next pilot, we will implement changes to the practice component of the intervention to encourage greater adherence to the intervention protocol. We will also explore adapting the intervention for in-class participation and online use to reduce any barriers to participation.

## Introduction

Approximately 40% of mortality risk is attributable to lifestyle [1]. For example, people who engage in a lot of sedentary behavior have a higher all-cause mortality risk when they also have lower physical activity levels [2]. Yet, behavior change is difficult. Talking with an empathetic health-care practitioner is one method that may encourage behavior change [3]. People may have multiple potential opportunities to talk with a health-care practitioner in the course of their lives, as health counseling is not limited to one discipline. Respiratory, Physical, and Occupational Therapy; Nurse Practitioner; and Kinesiology are just some of the disciplines that provide health counseling. However, sometimes these conversations are difficult for both parties [4]. Interventions that train health-care practitioners to sensitively approach conversations about health behaviors with empathy are essential for therapeutic engagement and effective person-centered goal setting. Understanding the feasibility, acceptability, and appropriateness of a promising empathy intervention is an important first step to developing such an intervention [5], and is the focus of the present research.

### Empathy

Despite the demonstrated effectiveness of health counseling, many clients have reported having difficulty communicating with health-care practitioners about health behaviors [6]. Health-care practitioners can feel that they lack health counseling skills [7]. Students of health-care fields in particular can feel awkward talking with clients [4]. In these situations, clients can feel unheard and perceive the practitioner as being paternalistic [4]. Evidently, providing students with opportunities to learn and practice their communication skills is critical.

An important feature of health behavior communication is empathy [8]: the multi-dimensional skill of appreciating and experiencing someone else’s thoughts and feelings [9]. Cognitive empathy (a.k.a. perspective-taking) is the *imaginative ability to put oneself in the mindset of the client* to understand their feelings and motives [10]. Health-care practitioners are not only seen as having better interpersonal skills when they perspective-take [11], they also convey that they care [12]. Furthermore, health-care practitioners who perspective-take have clients who are more satisfied [11] and more likely to adhere to a treatment plan [13] than those who do not. While people can be naturally empathetic, learning self-awareness and how to take others’ perspectives in a way that does not involve experiencing others’ emotions is important for avoiding burnout [14, 15]. Thus, perspective-taking interventions are important for students and practitioners.

#### Framework

**Fig. 1:**
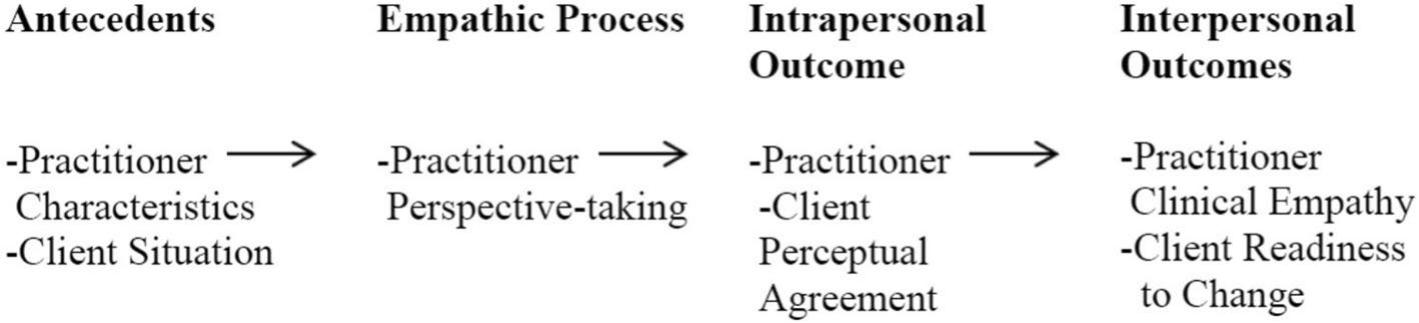
Adapted Davis’s (1994) organizational model on empathy

Davis’s (1994) empathy model (Fig. 1) illustrates how perspective-taking is a vital part of client-practitioner interactions [16]. The model identifies antecedents, mediation, and outcomes. Examples of antecedents include practitioner characteristics (e.g., mood, attitudes toward preventive counseling) and aspects of the client’s situation (e.g., the client’s type of health behavior). Perspective-taking is a mediating empathic process between these antecedents and intrapersonal and interpersonal outcomes. An example of an intrapersonal outcome includes client-practitioner perceptual agreement (i.e., the congruence between practitioner’s *inferences of* the client’s thoughts and feelings and the client’s *actual* thoughts and feelings about the client’s behaviors). An example of an interpersonal outcome includes the client’s readiness to change their physical activity level. As the model outlines, practitioner perspective-taking plays a central role in promoting outcomes that facilitate behavior change in clients.

Research in health-care supports parts of this model. For example, in regard to intrapersonal outcomes, perspective-taking is associated with client-practitioner perceptual agreement about at-risk health behaviors [7, 17]. In regard to interpersonal outcomes, perspective-taking promotes a supportive practitioner communication style and lessens judgment of others’ behaviors [18]. Thus, there is support for Davis’s (1994) model within the health-care context and implications for pilot feasibility testing an intervention based on it.

#### Teaching perspective-taking

Fortunately, students want communication skills training on how to deal with sensitive issues [19], and perspective-taking is a communication skill that can be taught [20, 21]. Students can improve empathic processes like perspective-taking when they practice self-awareness of personal values/emotions that can thwart empathy [22], receive instruction [23], and use video-feedback to self-evaluate [24]. For example, Fukkink and colleagues’ (2011) meta-analysis found that the aggregate effect of video-feedback on communication skills in a variety of professions was a medium-sized Hedges’ *g* effect size of 0.40 [24]. Students seem to appreciate the opportunity for self-reflection and self-growth during this formative time [25]. The inclusion of practicing self-awareness, instruction, and video-feedback in a perspective-taking intervention may build this important skill among health-care students.

### Present study

Despite the importance of perspective-taking in communication, this skill has not been well-developed in health counseling [26]. Providing students with practice opportunities and feedback specifically around perspective-taking represents an important component of evidence-based communication training. This study’s primary purpose was to assess the feasibility, appropriateness, and acceptability of a randomized perspective-taking intervention with students in health fields where conversations with patients about health behaviors may occur. Including a partial intervention condition at the feasibility stage provides a comparator group and an indicator of participation rates and quality of learning when the full intervention is not provided [27]. We draw upon the ORBIT model [28], to situate the present research within our larger intervention development efforts. ORBIT offers a guide for the development of behavioral interventions that target chronic disease prevention and treatment by outlining stages of intervention development and testing. The present work is in phase II (preliminary testing) b, (pilot feasibility work), which builds upon proof-of-concept research regarding the value of perspective-taking in health-care communication [7, 17, 18]. Pilot and feasibility work of this nature is recommended [5]; this study helped us prepare for unexpected events, and conceive of mitigating factors, for a future definitive trial testing the efficacy of our planned perspective-taking intervention [27]. We hypothesized that the present study would be feasible, appropriate, and acceptable according to study-specific criteria.

## Method

Materials, full measure details, and a procedural flow chart outlining the study’s phases are in Additional file 1. We did not obtain consent from participants to share their data with other researchers. The only major change we made after the trial commenced was to the eligibility criteria. Respiratory Therapy was added as an eligible program, because it was identified as a relevant program, additional funding was obtained, and course instructors were interested in having their students participate. This research received ethics board approval from the investigators’ university E2018:008 (HS21516). This randomized, mixed-methods pilot and feasibility study was registered at ClinicalTrials.gov.

### Participants and recruitment

To be included, participants needed to be a student at the investigators’ university, and they needed to have completed course content on behavior change communication. We aimed to recruit students from each of the following programs: Respiratory, Physical, and Occupational Therapy; Nurse Practitioner; and Kinesiology and Recreation Management. Within these programs, we targeted courses that had a behavior change communication unit. The study was embedded within existing courses for all participating programs except Occupational Therapy. When the study was embedded within a course, an alternative assignment was also made available for students who chose not to participate in the research. Occupational Therapy instructors invited us to circulate invitations to their students instead of embedding it because there was no capacity to do so. Providing two different recruitment methods allowed us to compare them for feasibility. Participants were recruited by research assistants (RAs) who explained the study in class after the class had completed their behavior change communication unit. Interested participants contacted an RA. In the event that a student participated in the study more than once, any data beyond the first participation were excluded.

### Randomization and masking

Upon study enrollment, an RA randomly assigned participants to either a full intervention (FI; perspective-taking workshop plus independent practice plus video-feedback) or partial intervention (PI; video-feedback alone) condition using a 1:1 allocation ratio in Excel. In-lab RAs were not masked to study condition. The client-actors that participants interacted with were masked to condition. Investigators were masked while determining participants’ perceptual understanding scores (i.e., how accurate participants were at inferring the client-actor’s thoughts and feelings).

### The intervention

The intervention description is informed by the TIDieR checklist [29]. This student practitioner perspective-taking intervention compares the outcomes where some learn about and practice perspective-taking and others do not. All participants received at least a partial intervention through receiving video-feedback on a dialogue with a client-actor (see Procedure for more details). As part of the full intervention, in their own time and on their own, participants in the FI condition completed a 15-min online workshop focused on self-awareness and perspective-taking (full workshop materials are not currently publicly available). Specifically, the text-based workshop was delivered as a questionnaire through SurveyMonkey.com and took participants through seven steps: (1) Introducing emotional and cognitive empathy; (2) Practicing self-awareness through recognizing that people have diverse viewpoints, and entering a health risk behavior that they have trouble understanding and that affects how they interact with or support others into the survey program; (3) Introducing perspective-taking; (4) Identifying “cues” as motivators for the health risk behavior and entering them into the survey program; (5) Practice instructions and instructions on validating inferences about how the other views the health risk behavior; (6) An overview of what to expect for the in-lab session; and (7) Summarizing the workshop and practice tips. FI participants were asked to independently practice perspective-taking about a health risk behavior with a family member/friend using a practice sheet over a few days to two weeks (see Additional file 1). PI participants were provided a link to the perspective-taking workshop after the study was complete. The workshop and homework materials were developed by health-care professionals with university education. The RAs were paid, health-care students who were recruited for the purposes of the study.

### Feasibility outcomes

Feasibility outcomes were informed by Consolidated Standards of Reporting Trials guidelines [30] and guidelines provided by Thabane et al. (2010) [27]. We used qualitative and quantitative methods to assess feasibility outcomes. Outcomes addressed recruitment, the protocol, and measure appropriateness.

### Recruitment

For recruitment, we were interested in recruitment time, recruitment rates, instructor interest (which influenced access to participants), and participant characteristics. Given previous experience [4], we set a recruitment rate goal of 85–95% per course when the study was embedded within a course and 5–10% when it was not, as well as a recruitment time goal of two years. We anticipated that obtaining equal proportions from each program would be difficult, because (a) we would be approaching individual classes in different programs; (b) class sizes vary; (c) fitting such a study into a course schedule could be difficult; (d) autonomy over course syllabi varies; and relatedly (e) being able to embed the study varies. Thus, we aimed for 10–15 participants from each program as our success criteria (50–75 participants total).

#### The protocol

For the protocol to successfully progress to the definitive trial, we were interested in workshop completion, practice duration, lab session length, whether participants adhered to the dialogue session instructions, and employee training feasibility.

Our goal was to provide 1–2 weeks of time between study enrollment and the lab session. We planned to evaluate whether this was feasible in terms of study scheduling, as well as whether this was adequate time for FI participants to complete the workshop and practice perspective-taking (e.g., is 1–2 weeks enough time for busy students to learn and practice?). To determine whether we met this goal, we checked workshop completion logs and analyzed exit interview data. To determine feasibility of scheduled lab session length, we aimed to have sessions completed within two hours. To determine whether we met this goal, ML conducted a random check of the field notes. She examined six sets of five interviews and then two more interviews, for a total of 32 interviews. To determine whether students adhered to the directive to avoid planning for change, we analyzed client-actors’ exit interviews for whether participants were “fix focused,” i.e., initiated behavior change recommendations. We aimed for an adherence rate of 90%. We set out to complete the client-actors’ and research assistants’ training in five hours. We evaluated practice sessions, five interviews, field notes, and exit interviews to assess readiness and need for additional employee training.

#### Appropriateness

We investigated the appropriateness of our measures by examining the means to ensure that they were not extreme [31] and evaluating the missing data to ensure that there were no more than 5% missing for each measure [32]. We set a cut-off value of 0.70 for Cronbach’s alpha [33]. Meeting each of these was essential for the successful progression to the definitive trial. We also analyzed the open-ended questions in the preliminary questionnaire to ensure that the questions were clear. Open-ended questions asked participants their gender, age, ethnicity, year in program, the type of communication training that they had received on how to communicate with clients (if applicable), the type of training that they had received on how to talk with clients about health risk behaviors for chronic illness (if applicable), and the type of health risk behavior that they were looking to change about themselves. We determined the clarity of the questions by looking at whether responses answered the question in the way we intended. For example, for communication training type, we were looking for whether participants mentioned training such as motivational interviewing. Responding with “E-mail” would be an example of needing to clarify the question for the definitive trial.

### Secondary outcomes

We observed instructor interest rates and integration rates for embedding the study in their course to get a sense of the feasibility of recruiting through courses, whether embedded or not. We also observed participants’ characteristics to better understand what we might expect for the definitive trial and because practitioner’s characteristics are a component of Davis’s (1994) empathy model (Fig. 1) [16]. Specifically, we collected demographics including age, gender identity, and health risk behaviors, as well as participants’ self-reported communication training experience. We would like to compare FI participants to participants who have existing counseling training in the definitive trial. Thus, understanding previous communication training experience in the target population was helpful for this goal. Additionally, we were interested in the acceptability of the study.

#### Acceptability

Acceptability was assessed through examining responses to exit interviews and field notes (see Analysis below for additional details). We examined the acceptability of the workshop, dialogue, and video-tagging exercise. We also wanted to understand how PI participants approached the dialogue to know what to expect for the definitive trial.

### Procedure

This parallel two-arm mixed-methods trial involved four phases (see procedural flow chart in Additional File 1).

#### Phase 1: workshop

As previously mentioned, in the FI condition, participants completed a consent form before completing an online workshop focused on self-awareness and perspective-taking. FI participants were sent daily reminder emails to promote intervention adherence. PI participants were provided with a link to the perspective-taking workshop after the study was complete, and completed their consent form prior to completing the preliminary questionnaire.

#### Phase 2: preliminary questionnaire and video-recorded dialogue

Approximately two weeks after enrollment, individual FI and PI participants engaged in a video-recorded perspective-taking exercise with a client-actor and completed other study measures in the lab. The communication lab contained private home-like rooms with built-in cameras and microphones in a local hospital. Participants first completed an online questionnaire that included demographic questions, the Positive and Negative Affect Scale [34] to measure mood, the Preventative Medicine Attitudes and Activities Questionnaire [35] to measure knowledge about lifestyle counseling, and the Consultation and Relational Empathy questionnaire (CARE; [36, 37]) to measure their perceptions of how the client-actor would perceive them in the upcoming video-recorded perspective-taking exercise. Next, the RA shared with the participant that the client-actor was moderately ready to change their physical activity level based on the Readiness to Change Ruler [38]. The RA instructed the participant that they were not to make a plan of action with the client to change the client’s behavior, and instead, they were to focus on listening to what the client had to say. FI participants were instructed to implement the perspective-taking technique that they had learned through the workshop for the exercise. PI participants were instructed to be aware and mindful of the approach that they took to seek understanding. Participants then had a private, face-to-face, video-recorded, 10-min dialogue with a client-actor about the client’s physical activity level.

Each actor was trained to act as a client with moderate readiness to change and low physical activity levels (see Additional file 1 for their script). The client-actor was one of two hired and trained men.^1^They were instructed to approach each session from this common situation. Further, we selected client-actors who truly had low physical activity levels and had moderate readiness to change so that they could draw on real-life experience. The common behavioral focus on physical activity and the common client situation for all participants enhanced internal validity (i.e., by reducing variability that could be introduced if participants focused on different behaviors/worked with clients that were very different).

#### *Phase 3*: *video-tagging exercise*

Immediately following Phase 2’s video-recorded dialogue**,** the client-actor and participant were separated to different rooms. The client-actor completed a video-tagging exercise using Studiocode™ (later called Vosaic™) software on a MacBook laptop. With this software, the client-actor viewed the dialogue video, stopped it each time they recalled experiencing a distinct thought or feeling, and recorded (or ‘tagged’) that thought or feeling in that instance. Meanwhile and in their separate room, the participant was invited to quietly watch the dialogue video on an iMac. After asking the participant how they felt seeing themselves in the video, the RA demonstrated the video-tagging exercise using a pre-recorded video of a tagged dialogue between one of the investigators (ML) and her undergraduate nursing student (not a study participant) who posed as a client concerned about his poor diet as a health risk behavior. By this time, the client-actor was done with their video-tagging exercise, and it was the participant’s turn to view their video again and infer what the client-actor was thinking or feeling at each of the previously tagged instances. The video-tagging exercise assessed how accurate the participant was at perspective-taking [39].

#### *Phase 4*: *final questionnaire and exit interview*

After the video-tagging, the participant and the client-actor separately completed an online questionnaire followed by a 30-min exit interview with an RA. This online questionnaire invited participants to complete the CARE again, this time assessing their perceptions of how the client-actor perceived them in the video-recorded perspective-taking exercise. The client-actors also completed a version of the CARE, in which they provided their perceptions of the participant. Additionally, the client-actors completed a measure to determine whether their readiness to change their level of physical activity differed from the moderate ratings provided at baseline.

The exit interview was guided by a script of open-ended questions. Responses were captured using hand-written or typed notes. Participants and the client-actors were interviewed separately. They were both asked to independently describe the participant’s approach. Participants were also asked about their perceptions of the study phases to help the researchers further develop the intervention. The RA’s notes of these interviews were used to assess the acceptability of the workshop, video-recorded dialogues, and video-tagging exercises. Given that participants were instructed not to make a plan of change for the client, client-actors’ interviews were used to assess whether participants adhered to this instruction.

After the study was completed and while masked to study condition, two investigators (ML and SS) and a trained coder individually rated 25 randomly selected participants’ video-tagged responses using the investigator-developed similarity rating tool to determine participant perceptual understanding scores (see Additional file 1 for more details). They then met to compare their ratings and determine participants’ perceptual understanding scores. A discussion followed whenever differences occurred, resulting in consensus and 100% interrater reliability on the rating tool. The trained coder then completed the ratings for the rest of the participants using the tool. One investigator is a health-care professional with university education, and the other is a non-health-care professional with university education who teaches about health. The coder was a paid health-care student who was recruited for the purposes of the study.

### Sample size

We aimed to recruit 10 to 15 students from each program (50–75 participants total). This target sample size aligns with guidelines for determining sample sizes for pilot/feasibility studies. For example, our target sample size of 50–75 participants (at least 10 per program across 5 programs) allows us to estimate a recruitment rate of 85% to within a 95% confidence interval of ± 10% [40]. We reasoned that if we happened to get a sample size larger than this, then that would bolster our confidence in (a) interest in the intervention and (b) the feasibility of recruiting an adequate sample size for the definitive trial.

### Data analysis

We used SPSS v.17.0 to analyze the quantitative data. We used frequency counts and percentages to describe the sample, and assess recruitment and protocol outcomes. The mean and range were used to describe age. To assess psychometric outcomes, we calculated (a) means, standard deviations, and ranges to determine floor and ceiling effects; (b) the percentage of missing data; and (c) Cronbach’s alpha. To determine the acceptability of the intervention, two new RAs independently examined exit interview responses using manifest content analysis and constant comparison techniques to identify, code, categorize, classify, and label the primary patterns in the data [41]. With this analysis, we captured what students said, and used participants’ own words for codes and themes when possible to ensure that we stayed close to the data. For example, “it was kind of awkward” was coded as “awkward.” Disagreements were resolved through discussion. Code categories were organized according to themes. For example, codes for the video-recorded dialogue being “good,” allowing for “client connection,” the participant being “non-committal,” and finding the dialogue “awkward” were combined under the theme “dialogue experience.” Themes were grouped by intervention phase for reporting. Dependability was enhanced by having the RAs, and two investigators (ML and SS) regularly review the coding process and agree on analyses. We enhanced transferability by referring to the written observations made by the RAs who conducted the study when coding the interview responses [42]. The new RAs were also paid, health-care students who were recruited for the purposes of the study.

## Results

### Feasibility

Data were collected between February 2018 and December 2019. Data collection ended because we reached the two-year time limit and had attempted recruitment from all target units.

### Recruitment

**Fig. 2:**
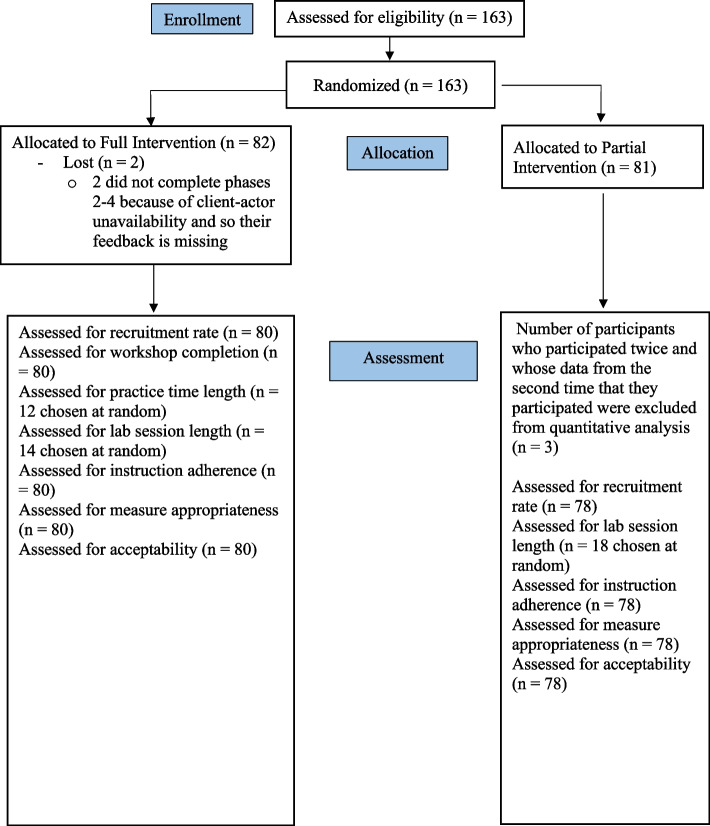
CONSORT diagram, flow chart of students in the Full and Partial Intervention conditions

We recruited 163 participants (Fig. 2). Three PI participants participated twice, and so data from their second session were excluded. Two FI participants were lost to follow-up, because of scheduling conflicts. We met our goal of completing all recruitment within two years (Table 1). We fell slightly short of our recruitment rate goal of 85–95% when the study was embedded within a course. We did meet this goal for Physical Therapy (85%; 85/100 students) and Kinesiology (92%; 22/24 students; note: there were three participants in this course who were from other programs). However, we fell short for Respiratory Therapy (83%; 25/30 and the Nurse Practitioner program (80%; 24/30 students). We also fell slightly short of our non-embedded recruitment goal (and, relatedly, our success criteria of 10–15 participants from each program) as only 4% (2/50 students) from Occupational Therapy participated. Apart from the recruitment goals, as mentioned, three students participated twice indicating student interest in the study.

**Table 1:** Feasibility study criteria and whether these were met

Variable	Results	Criteria	Criteria met	Criteria not met
Recruitment				
Recruitment time	February 2018–December 2019	Within 2 years	x	
Recruitment rate when embedded within a course	Physical Therapy: 85% Respiratory Therapy: 83% Kinesiology: 92% Nurse Practitioner: 80%	85–95%	Met for Physical Therapy and Kinesiology programs	Short for Respiratory therapy and Nurse Practitioner programs
Recruitment rate when not embedded within a course	Occupational Therapy: 4%	5–10%		x
Program recruitment	Physical Therapy *n* = 87 Respiratory Therapy *n* = 28 Kinesiology *n* = 19 Nurse practitioner *n* = 24 Occupational Therapy *n* = 2	10–15 participants per program	Met for Physical Therapy, Respiratory Therapy, Kinesiology, and Nurse Practitioner programs	Short for Occupational Therapy
Protocol				
Workshop completion	1 did not complete and 3 partially completed the workshop	100% completion		x
Practice time length	Often 1 week Some did not practice	1–2 weeks		x
Lab session length	Mean = 2 h; most completed the lab session in less than 2 h; 6 completed it in 2 h and 1 completed it in 2 h, 3 min	2 h	x	
Adherence for not making a plan of action with the client-actor	94%	90%	x	
Employee training	5 h	5 h	Met for most employees	Second actor needed additional guidance
Psychometric outcomes				
Extreme means	Most means were not extreme	No evidence of floor or ceiling effects	Met for most variables	The Importance subscales of the Preventative Medicine Attitudes and Activities Questionnaire displayed ceiling effects
Missing data	Nothing less than 5%	No more than 5% for each measure	x	
Cronbach’s alpha	All scales and subscales had Cronbach’s alpha values greater than .70, except one	Cut-off of .70	Met for most variables	Lifestyle Counseling Effectiveness subscale of the Preventative Medicine Attitudes and Activities Questionnaire’s *α* = .51

### The protocol

For the protocol to successfully progress to the definitive trial, we were interested in workshop completion, practice duration, lab session length, adherence rates for the instruction to avoid planning for change, and employee training feasibility. Looking at the workshop access log data, we learned that some FI participants did not complete the workshop. Participants often had one week between study enrollment and the lab session. Our aim was to provide one to two weeks of workshop completion and practice time, with daily practice reminders. Discussions with RAs indicated that it was possible the reminder was not sent on a small number of occasions. From exit interviews, we learned that some participants did not practice. These participants mostly said that they just reviewed the intervention materials (Table 2). A few said that they did not have anyone to practice with because they did not see anyone during the practice period or did not have an opportunity to practice. For example, one participant said, “I live alone and I’m not from here, but I did read through them.”

**Table 2:** Participant qualitative responses toward the intervention

Intervention phase and theme	Definition	Quote
All phases—Welcoming environment	Student made a comment about the study environment being a comfortable, welcoming, or an overall positive experience. Student mentioned that they liked the study or certain aspect(s) of the study (i.e., they thought the study was beneficial for health-care providers or they enjoyed the tagging exercise)	Field note quote about an FI participant: “he liked the space and that it simulates a natural environment.”
Phase 1: Workshop—Review only	FI participant indicated that they reviewed the workshop materials	“Mostly reviewed it.” – FI participant
Phase 2: Video-recorded dialogue—Dialogue experience	Student commented on their experience of the 10-min dialogue/interaction with the client	-
Good	Student stated experience was good or went well	“Good” – PI participant
Allows for client connection	Student felt that they were able to better connect, relate to, or build a rapport/therapeutic relationship with the client	“It makes me feel a little more connected even though I don’t think I strongly connected with him I think the approach does help you connect better.” – FI participant
Non-committal	Student stated experience was okay, fine, not bad, “I don’t know”, or used sarcasm to answer	“It was okay. I had general nerves, but I think it was okay.” – FI participant “It was clinical because I knew I was being filmed. I wanted to act like a professional and not seem too friendly. For that reason, I did not do too much small talk.”—PI participant
Awkward	Student stated experience was awkward or not good	“it was kind of awkward.” – FI participant
Phase 3: Video-tagging exercise—Initial video-feedback experience	Student commented on their initial experience watching themselves during the 10-min dialogue via video-feedback	-
Good	Student stated experience was good or went well	“It was fine.”—FI participant One participant said that she liked the feedback from the tagging; “…trying to determine if the interaction was going positively or negatively.” – PI participant
Non-committal	Student stated experience was okay, fine, not bad, or used sarcasm to answer	“We had to do it in class (film yourself) so I’m used to it now.” – FI participant “I’m used to seeing myself in video, as arrogant as that sounds.” – PI participant
Awkward	Student expressed discomfort at seeing themselves in the interaction, stating that it was weird or cringy	“you know in sitcoms when you know an embarrassing part is coming up and you just wanna turn it off or skip ahead? Yeah that’s how I felt watching myself” – FI participant
Difficulty with tagging	Student thought the tagging exercise was difficult or challenging to do	“A little more challenging than I thought. Having to disregard my opinions to figure out what he’s thinking or feeling.” – FI participant
Thought vs. feeling	Student expressed that the tagging exercise was difficult because it was hard to differentiate between a thought and a feeling	Field note indicated that an FI participant thought it was hard to distinguish between thoughts and feelings

*FI* full intervention, *PI* partial intervention

We were able to meet our goal of having sessions completed within two hours. The check of a random sample of field notes indicated that most participants (25/32) spent less than two hours in the lab; six participants spent two hours and one participant spent two hours, three minutes in the lab. Client-actor exit interview responses indicated that we were able to meet our goal of having an adherence rate of 90% for not making a plan of action, as only 10 participants (out of 158) were reported to be “fix focused.” We were also able to meet our goal of training RAs and the first actor in five hours. However, the second actor was trained by the first actor and needed additional guidance as the study progressed. For example, ML reminded the second actor twice to follow the script after reviewing video recordings.

### Appropriateness

To assess the suitability of study measures for progressing to the definitive trial, we calculated means, standard deviations, and ranges to determine floor and ceiling effects; the extent of missing data; and internal consistency (Table 3; see Additional file 1 for results split by condition). Formal tests of statistical significance were not carried out, because the focus of this pilot was on assessing feasibility [27]. The Importance subscales of the Preventative Medicine Attitudes and Activities Questionnaire displayed a potential range restriction with most participants reporting that asking and counseling about health risk behaviors was very important. Missing data was minimal with all measures having 2% or less data missing. All scales and subscales also showed Cronbach’s alpha values greater than 0.70, except the Lifestyle Counseling Effectiveness subscale of the Preventative Medicine Attitudes and Activities Questionnaire (*α* = 0.51). Removing items on this subscale did not significantly improve the alpha. We return to this questionnaire in the discussion section.

**Table 3:** Participant and client-actor descriptive statistics

Measure (possible range)	*n*	Mean (SD)	Range	Missing data	Cronbach’s alpha
Participant					
PMAAQ Behavior Change Effectiveness (1–5)	156	2.69 (0.64)	1.29–4.14	< 2%	.83
PMAAQ Lifestyle Counseling Effectiveness (1–5)	156	3.10 (0.40)	2.08–4.08	< 2%	.51
PMAAQ Importance Ask (1–4)	157	1.40 (0.41)	1–3	< 1%	.82
PMAAQ Importance Counsel (1–4)	156	1.38 (0.41)	1–3	< 2%	.83
Positive Affect (10–50)^a^	158	26.31 (7.09)	11–43	0%	.90
Negative Affect (10–50)	158	15.57 (4.04)	10–32	0%	.75
Pre-Interview CARE (9–54)^a^	158	28.82 (5.60)	9–44	0%	.91
Post-Interview CARE (9–54)^a^	158	26.97 (5.60)	13–41	0%	.87
Perceptual Understanding (0–100)^a^	158	50.99 (18.18)	0–85.71	0%	N/A
Client-actor					
CARE (10–60)^a^	158	26.23 (6.25)	12–44	0%	.92
Readiness to Change (0–10)^a^	158	5.61 (0.86)	2.50–8.50	0%	N/A

*PMAAQ* Preventative Medicine Attitudes and Activities Questionnaire, *CARE* Consultation and Relational Empathy.

^a^Higher values are better

In regards to question clarity, examining responses to the two open-ended questions on the demographic tool about the type of communication training received to communicate with clients and to talk with clients about health risk behaviors for chronic illness indicated that these questions either needed re-wording and/or examples. Participants either wrote about specific courses where they received communication training (e.g., “Health Education labs”) or wrote about a counseling approach (e.g., “motivational interviewing”). Other open-ended questions on the demographic tool appeared clear.

### Secondary outcomes

Five instructors were directly approached about including the study in their course. These instructors were in Occupational Therapy, Physical Therapy, and Nurse Practitioner programs. SS (co-investigator) included the study as part of her course in Kinesiology and Recreation Management. For SS, steps were taken to ensure we maintained ethical standards by having SS leave the room when recruitment occurred, so that students did not feel pressure to participate. Students in the Kinesiology and Recreation Management who did not agree to participate (*n* = 1) were given the option to write a paper that an RA graded with a provided rubric to ensure that SS did not know who participated and who did not. We were approached by the College of Medicine; however, they were unable to fit the study into their program. Part way through data collection, we were also approached by the Respiratory Therapy Department. We sought, and received, additional funding to be able to include these students in the study. Moreover, there was the capacity to embed the study in all participating programs except Occupational Therapy.

### Participant characteristics

We examined participants’ characteristics to understand what to expect for the definitive trial. Most participants (62%) identified as women, and most participants (58%) were of European origins in their mid-20 s (*M*age = 25.94, *SD*age = 4.45 years; Table 4). Most participants (85% FI, 91.1% PI) reported receiving previous communication training, and most received training on how to talk with clients about health risk behaviors for chronic illness (68.8% FI, 69.6% PI). About half of the participants (53.8% FI, 53.2% PI) indicated that they themselves had health risk behaviors they would like to change.

**Table 4:** Participant demographics

Variable	Full intervention (*n* = 80)	Partial intervention (*n* = 78)
Gender (open-ended)	Men = 32 (40%) Women = 47 (61.8%)	Men = 28 (35.9%) Women = 50 (64.1%)
Age (range to choose 0 -100 years)	Mean = 25.21 (21—39)	Mean = 26.69 (20—42)
Ethnicity (open-ended)	Asian = 26 (32.5%) African = 2 (2.5%) Canadian = 2 (2.5%) Caribbean = 1 (1.3%) European origins = 47 (58.8%) Latin/Central/South American = 1 (1.3%) North American Indigenous = 1 (1.3%)	Asian = 16 (20.5%) African = 4 (5.1%) Canadian = 7 (9%) Caribbean = 1 (1.3%) European origins = 44 (56.4%) Latin/Central/South American = 2 (2.6%) North American Indigenous = 1 (1.3%) “Mixed” = 2 (2.6%) Prefer not to say = 1 (1.3%)
Program (Kinesiology, Occupational Therapy, Nurse Practitioner, Physical Therapy, Other-specify)	Kinesiology = 9 (11.3%) Occupational Therapy = 1 (1.3%) Nurse Practitioner = 10 (12.5%) Physical Therapy = 44 (55%) Other = 16 (20%) - Respiratory Therapy = 15 (18.8%) - Recreation Management and Community Development = 1 (1.3%)	Kinesiology = 10 (12.8%) Occupational Therapy = 1 (1.3%) Nurse Practitioner = 14 (17.9%) Physical Therapy = 41 (52.6%) Other = 12 (15.4%) - Respiratory Therapy = 10 (12.6%) - Nutritional Sciences = 1 (1.3%) - Physical Education = 1 (1.3%)
Year in program (open-ended)	1 = 10 (12.5%) 2 = 58 (72.5%) 3 = 2 (2.5%) 4 = 7 (8.8%) 6 = 1 (1.3%) Missing = 2 (2.5%)	1 = 15 (19.2%) 2 = 48 (61.5%) 3 = 3 (3.8%) 4 = 5 (6.4%) 5 = 5 (6.4%) Missing = 2 (2.6%)
Type of health risk behavior participant wishes to change in themselves (open-ended)	Poor diet = 25 (31.3%) Smoking/Tobacco = 2 (2.5%) Lack of exercise = 18 (22.5%) Alcohol/drugs = 4 (5%) Sleep-related = 2 (2.5%) Anxiety and/or stress = 3 (3.75%) Other (e.g., screen time) = 1 (1.3%)	Poor diet = 23 (29.5%) Smoking/Tobacco = 2 (2.6%) Lack of exercise = 20 (25.6%) Alcohol/drugs = 2 (2.6%) Sleep-related = 8 (10.3%) Anxiety and/or stress = 4 (5.1%) Other (e.g., screen time) = 3 (3.9%)

### Acceptability

Analysis of the field notes and exit interviews revealed themes related to positive experiences and non-commitment to the process with each phase of the study (Table 3). One theme that emerged was on the welcoming environment. Some participants commented on how the lab environment was comfortable and welcoming. For example, field notes showed that one FI participant thought that the study was really well done; it was a very welcoming atmosphere, and he liked the living room where the dialogue took place.

Responses indicated that the dialogue experience was largely acceptable and a formative opportunity. Some participants had a positive experience, saying that they thought it was good and allowed for connection. For example, a PI participant said, “Yeah I think it gave me … I cared about what was going on with Kyle even though he is an actor.” Some were non-committal in their responses (e.g., an FI participant said, “it was okay I think”), whereas a few thought it was awkward. For example, an FI participant said that “It felt really awkward. I know we had a week to practice but it’s a new way of speaking and you always have to think about if you’re doing this properly. You’re thinking about the client and how he’s feeling but also if you’re doing it properly. It made things stilted, stuck and awkward and a lot more stuttering.”

Similar to the dialogue experience, the most common responses to the video-feedback experience were good, non-committal, and awkward. For example, an FI participant said, “Not bad. I actually impressed myself” and a PI participant said, “No one likes watching themselves.” A few participants had difficulty with perspective-taking. For example, one FI participant said, “A little more challenging than I thought. Having to disregard my opinions to figure out what he’s thinking or feeling.” Additionally, a small number had difficulty distinguishing between a thought and feeling, indicating that improvements to the video-tagging portion of the protocol could be made.

### Partial intervention participants’ approaches to seeking understanding in the dialogue

We wanted to understand how PI participants approached the dialogue to know what to expect for the definitive trial. To do so, we analyzed exit interviews and field notes for PI participants’ dialogue approaches. Analysis revealed that they employed a variety of techniques, including perspective-taking. Participants related to the client’s experiences by trying to take the client’s perspective and using active listening techniques. For example, one PI participant said, “I think as a student I can 100% relate to this situation.” They tried to convey warmth and use a beneficial line of questioning — for example, asking open-ended questions and reflecting back to the client what they heard. Outside of techniques that would have been learned in the workshop, there were PI participants who reported lecturing the client. These techniques were helpful to understand, because they indicated that participants who already had health counseling training were using some skills that were also taught to FI participants during the workshop. Knowing there were some PI participants who were lecturing the client indicated that there was still some room for improvement in physical activity counseling in this group.

## Discussion

The purpose of this randomized study was to assess the feasibility, appropriateness, and acceptability of a theoretically informed perspective-taking educational intervention in preparation for a definitive trial. The ultimate goal of the definitive trial will be to determine whether providing health-care students with instruction and practice in perspective-taking enhances their counseling competence. We learned that the study should be largely feasible, appropriate, and acceptable. However, there are some issues that have implications for progression to the definitive trial and are large enough to warrant conducting another pilot trial.

### Recruitment

Recruiting university students can be challenging [43]. We were able to meet several of our recruitment goals, but we fell short on some specific aspects of recruitment. We met our success criteria of 10–15 participants from each program with the exception of Occupational Therapy where we only recruited two participants. We attribute the low recruitment rate within Occupational Therapy to the fact that participation was not embedded within a course. Embedding within a course emphasizes the relevance of the training and is compelling enough for students to make time (so much so that three students participated twice). We are encouraged by the fact that all other programs were open to embedding the study within a course. In these courses, we surpassed the criteria of 10–15 per program for most programs which left us less concerned about falling slightly short of our goal of 85–95% participation within two of these courses. As such, we are confident that we will reach our recruitment goals to be able to do significance testing in the definitive trial. While future research will determine the minimally important difference, previous pilot research using a similar paradigm yielded an effect size (Cohen’s* d*) of 0.43 for practitioner CARE scores [4]. According to G*Power (version 3.1) [44], we will need 172 participants (86 participants per condition) to detect a Cohen’s *d* of 0.43 (80% power, *α* = 0.05). We will apply for funding that will allow for a slightly longer recruitment time to be able to reach this goal and will conduct another pilot with some recruitment changes (see Table 5 for suggested changes). While there were three participants from outside the target programs who participated due to their being in a targeted course, these students would have received the same counseling communication content within the course as their classmates. Thus, we believe that their data were still valuable and plan to continue to allow students enrolled in the target courses from outside programs to participate.

**Table 5:** Suggested changes for future trials

Suggestion
Continue to embed the study within a course
Explore adapting the intervention for online or in-class use
Recruit students who have not completed any health counseling if independent practice rates have not improved through alternative means
Extend the amount of time participants have to practice perspective-taking and have participants complete a daily log as well as implementation intentions to encourage practice
If using client-actors, train them directly, rather than have one actor train another

Despite Occupational Therapy instructors’ interest in the intervention, they were unable to embed the study within a course and warned us that their course is quite heavy for students. Medicine is another program that could not fit this study into their schedule. Thus, while instructors were interested in the study, enough that it spread through word of mouth, instructor interest was not always sufficient to help us meet our recruitment goals. Being able to fit the study into a course was important for helping us meet our goals and will be employed in the definitive trial. Telling instructors that participants valued seeing themselves and reflecting on their interaction, and giving instructors enough advanced notice so they can think about how to incorporate the study could be beneficial. Additionally, offering instructors flexibility in how to incorporate the study may help.

One such avenue we are exploring is giving students the opportunity to participate in the study during class time. It would require fitting it in the class schedule, but would take up less of students’ time. Those in the FI condition would do the workshop and practice perspective-taking before coming to class and those in the PI condition would complete the workshop after the class. Students would complete the video-recording, video-tagging exercises, and receive feedback with their selected dialogue partner in-class. The dialogue partner could be a client-actor or peer [45], because both types of partners are helpful for practicing communication skills. Students interested in participating in the study would receive the questionnaires. This would give them experience with the intervention’s components and help normalize research [46].

Given limitations with lab space and the COVID-19 pandemic, and to make in- and out-of-class experiences related to the study easier, we are also exploring providing the study online [47]. By doing so, we can increase who can participate, the number of participants, and the times when they can participate. While adapting the intervention can limit the current study’s generalizability, similar research on motivational interview training found that it is feasible to provide online communication training and virtual client interaction opportunities [48]. In their study, Oster and colleagues found initial evidence that counseling skills and attitudes toward behavior change counseling could improve after online training and interacting with a virtual client. Future research should compare a fully online experience to the existing experience to determine if they are equal or if one method is better.

Targeting courses and programs that had behavior change communication training was helpful for assessing the feasibility of comparing FI participants to PI participants in the definitive trial. Given that PI participants reported using techniques similar to those taught to students in the FI condition, it could be worth targeting students who have not completed any communication training to increase the likelihood that participants will practice perspective-taking after they receive training. Students who have not completed any communication training may be more interested in learning, practicing, and receiving feedback on perspective-taking skills than those who are farther along in their program. Some research indicates that this could be the case as nursing students who have not received training on how to discuss sensitive health issues and who lack exposure to clients who face sensitive issues express desire for such training and exposure [19]. As our current strategy of targeting courses with some communication training allows for sufficient recruitment rates, we will first focus on implementing protocol changes that reduce barriers to participants’ ability to practice before exploring changing our recruitment criteria.

### The protocol

We did not meet success criteria for workshop completion and practice time length. Results indicated that we should extend the practice time length to allow for additional opportunity to complete the workshop and practice. Students in the FI condition did not always participate in the workshop or practice perspective-taking, and final exams may have played a role for some. Avoiding testing during the final exam period, increasing the practice period, ensuring reminders are sent, and reducing barriers to completing these may be important. Writing intentions to implement a goal in the form of if–then statements may help students to identify opportunities and potential barriers. By forming implementation intentions, people are able to recall the intentions and automate their behavior [49]. There is extensive research on implementation intentions and their effectiveness in motivational psychology [49, 50]. Implementation intentions may be helpful whenever educators, researchers, and health-care providers offer an activity that people might need motivation to complete. For example, by specifying who they will practice with and when, future participants can be more intentional about their practice. They can also then contact the researchers if they discover that they need someone to practice with. Future research should assess whether daily logs and implementation intentions improve practice rates.

Results indicated that lab session length and in-lab instruction adherence goals were feasible. In terms of employee training goals, results indicated that these were largely feasible; however, changes should be made for training actors, if they are used. One of the actors was trained by the researchers and then trained their successor. The successor needed extended training, indicating that direct training from the researchers will be necessary. Watching a sample of actor videos and using them in training, as well as having practice sessions with the client-actors and RAs until satisfied with their performance is recommended.

### Appropriateness

Analysis of the open-ended questions on communication training indicated that future inquiry could ask participants to specify whether previous training included a communication or counseling course and to describe the counseling techniques, including empathic approaches. Providing an example may also help. For close-ended questions in this study, analysis indicated that the psychometric properties were adequate. However, the Preventive Medicine Attitudes and Activities Questionnaire did not meet success criteria. Internal consistency was low for the adapted Lifestyle Counseling Effectiveness subscale, and there was some restriction of range with this questionnaire. Previous research on the original measure found higher internal consistency for the Lifestyle Counseling Effectiveness subscale (*α* = 0.89) [35]. The low score may be due to the adaptations and the subscale appearing to capture multiple concepts. We plan on retaining this adapted questionnaire because we believe it will explain variance in the outcome measures. However, we will perform confirmatory factor analysis and reliability analysis with a larger sample. If model fit is poor and internal consistency is low in the future trial, then we will omit the subscale from the analysis. If range restriction is replicated in future work, we will adjust correlations to account for the restriction [31].

### Acceptability

Instructors in the present study were enthusiastic about including the intervention and reported that their students enjoyed having the study as part of a course. Participants generally valued being able to practice dialoguing and receive video-feedback. Together, this indicates that the protocol was acceptable enough to proceed with another pilot.

Participants’ responses indicated that our next pilot should incorporate some changes to improve practice feasibility and the tagging experience. Feedback on the practice period indicated that participants in the definitive trial should have more time to practice, and have the resources to encourage practice (see Table 5). Allowing more time for practice may reduce students’ self-consciousness during the dialogue as well. Replicating other research, feedback on the video-tagging experience indicated that a small number of participants struggled with differentiating thoughts and feelings [4]. Participants received a sheet that described the differences between a thought and feeling, and one that is positive or negative. It is possible that participants struggled with this because everyday discourse often intermixes thoughts and feelings. For example, a person might say “I feel like that’s not true,” when it is technically correct to say “I think that’s not true.” While the similarity rating tool is based on research in social psychology [39] and refined in nursing [4], it may be necessary for future research to explore omitting the thought versus feeling distinction. Furthermore, we have since added an option for a neutral label to better capture instances where the thought or feeling is neutral.

## Strengths, limitations, and generalizability

This study takes the next logical step from proof-of-concept work to pilot feasibility testing, in an effort to build evidence for a future definitive trial testing a promising theory-based intervention. A strength of this study is that we learned that students and instructors in a variety of disciplines were interested in learning more about empathy. This finding has implications for extending perspective-taking interventions into diverse disciplines and for the definitive trial as it indicates potential feasibility. A strength of this intervention’s method is that it provides opportunities for practitioners to practice and receive feedback about perspective-taking specifically, without trying to elicit talk of change. Taking a step back and listening to clients helps clients feel understood [51, 52]. Motivational interviewing is an approach to having conversations about change that emphasizes accurate empathy (akin to perspective-taking) through encouraging practitioners to strategically listen and reflect [53]. Many students in the current study mentioned learning this approach. However, this widely used counseling approach also focuses on eliciting talk of changing behavior [54]. Instructors who teach motivational interviewing may consider adding practice and feedback opportunities where students do not elicit talk of change to their curriculum.

A limitation to this study is that an RA generated the randomization list using Excel and this same RA assigned participants to conditions. This method could have allowed the RA to manipulate allocations, though we do not suspect this to be the case. For our definitive trial, we will have a statistician not involved in the day-to-day operations of the study create the randomization list and have research staff assign participants to the next space in the list. Another limitation to the study was that the second actor behaved inconsistently across participants and was reminded twice to follow the script. As previously noted, when including client-actors, watching a sample of actor videos and using them in training, as well as having practice sessions with the client-actors and RAs until satisfied with their performance will be employed.

In terms of generalizability, this feasibility study was conducted at one Canadian university before COVID-19 restrictions were in place. Additionally, we focused on health-care students who had received some previous communication training. Results may be different if we had targeted seasoned health-care practitioners or students who had not received communication training. Therefore, generalizability to other settings, times, and samples may be limited. However, we expect our findings around practice adherence and actor training to be generalizable, and helpful to other researchers.

## Conclusions

Interdisciplinary collaborations are essential for furthering education and research on commonly faced issues. One such issue faced by health-related disciplines is effectively counseling individuals about health behavior change. To effectively counsel, accurately understanding others and conveying that understanding is important. The present pragmatic feasibility trial on teaching perspective-taking skills to health-care students indicated that the protocol is acceptable and largely feasible. However, we suggest some changes to potential method of delivery (e.g., in class or online), practice length, and employee training that warrant conducting another pilot. Taken together, this research provides evidence of a promising method for teaching perspective-taking to effectively counsel individuals about health behavior change.

## Supplementary Information

Additional file 1. Flow chart and study materials

## Data Availability

The data are not publicly available because we did not get permission from the participants. Most materials can be found in Additional file 1.

## Abbreviations

RA: Research Assistant
FI: Full intervention
PI: Partial intervention
CARE: Consultation and Relational Empathy questionnaire

## Footnotes

1 Both actors were men to help with consistency.
