# Supplementary material for: A randomized pragmatic feasibility trial to promote student perspective-taking on client physical activity level: a collaborative project
Source: Pilot Feasibility Stud. 2024 Sep 28;10:123. doi: 10.1186/s40814-024-01547-8 (PMC11437983; doi:10.1186/s40814-024-01547-8)
Supplement: Supplementary file 1 — Additional file 1. Flow chart and study materials [file 40814_2024_1547_MOESM1_ESM.docx]

**Online supplemental materials for “A randomized pragmatic feasibility trial to promote student perspective-taking on client physical activity level: A collaborative project”**

Flow Chart of Study Procedures 2

Materials 3

Daily Reminder Emails and Study Session Information Email 3

Practice sheet for FI participants 4

Measures 8

Client-actor script 12

Video-Tagging Exercise – Single User Rating Sheet 15

References 20

# Flow Chart of Study Procedures

Full Intervention

Randomization

Participant

CARE questionnaire

Exit Interview

Final Questionnaire

Participant

CARE questionnaire

Exit Interview

Final Questionnaire

Video-tagging exercise

Partial Intervention

Client-actor

CARE questionnaire

Readiness Ruler

Exit Interview

Client-actor

CARE questionnaire

Readiness Ruler

Exit Interview

Video-tagging exercise

Video-recorded dialogue with client-actor

Preliminary Questionnaire

Consent form; Demographics; Positive and Negative Affect Scale; Preventative Medicine Attitudes and Activities Questionnaire; Consultation and Relational Empathy questionnaire

--

--

Phase 2

Video-recorded dialogue with client-actor

Preliminary Questionnaire

Demographics; Positive and Negative Affect Scale; Preventative Medicine Attitudes and Activities Questionnaire; Consultation and Relational Empathy (CARE) questionnaire

1-2 weeks of at-home practice using practice sheet

Phase 1

Consent form; Online workshop on perspective taking and the empathic process

Phase 4

Phase 3

Debrief then access to perspective-taking workshop

Debrief

# Materials

## Daily Reminder Emails and Study Session Information Email

A version of the following email was sent to participants daily in the Full Intervention (FI) group from 1 week prior to study session.

Subject line: Physical activity communication study practice reminder

“Hello,

This is a simple reminder for you to practice your perspective-taking today with a friend or family member before your session on [insert study session date and time here] at [location].

If for whatever reason you need to reschedule, please contact X at XXXX or reply to this email.

Regards,

The research team”

A version of the following email was sent to participants in the Full Intervention group 2 days prior to study session.

Subject line: Study session details and physical activity communication study practice reminder

“Hello,

This is a simple reminder for you to practice your perspective-taking today with a friend or family member before your session on [insert study session date and time here].

Location

Please arrive at the lobby of the [location]. [Location] building is a brown brick building (5 stories) annexed to the hospital on the north side. You can enter the building from either west or east side sliding doors. I will come down to the lobby to meet with you at your scheduled time for the session to bring you up to the x Lab. If something unforeseen arises and/or you are running late, feel free to text or call me on my cell (x) or at the lab (x).

Getting here

There is there is free 2-hour parking along [location]. There are also several busses that either come to the [location] or within walking distance (e.g., 21, 22).

We look forward to seeing you soon!

Regards,

The research team”

A version of the following email was sent to participants in the Partial Intervention (PI) group 2 days prior to study session.

Subject line: Study session details

“Hello,

Location

Please arrive at the lobby of the [location]. [Location] building is a brown brick building (5 stories) annexed to the hospital on the north side. You can enter the building from either west or east side sliding doors. I will come down to the lobby to meet with you at your scheduled time for the session to bring you up to the x Lab. If something unforeseen arises and/or you are running late, feel free to text or call me on my cell (x) or at the lab (x).

Getting here

There is there is free 2-hour parking along [location]. There are also several busses that either come to the [location] or within walking distance (e.g., 21, 22).

We look forward to seeing you soon!

Regards,

The research team”

## Practice sheet for FI participants

**Homework Exercise Hand-out for Students**

**Empathy Practice Exercise**

Over the next week, I want you to practice noticing your friend’s or family member’s health risk behaviour and thinking about what motivates him or her to engage in that behaviour.

How? Just as we practised today:

**Step 1. Realizing your viewpoint may not be the same as the family member’s or friend’s viewpoint on the health risk behaviour.**

Identify your own viewpoint. Think about and identify your own positive and negative thoughts, feelings, beliefs, preferences, values, and judgments toward the health risk behaviour.

Being aware of your personal viewpoint will help you to engage in Step 2.

**Step 2. Taking the family member’s or friend’s viewpoint NOT your viewpoint.**

The approach you are to practice over the next week is to focus on and imagine how YOUR FAMILY MEMBER OR FRIEND is feeling about his or her health risk behaviour, NOT FROM YOUR POINT OF VIEW, but from your family member’s or friend’s point of view.

Another way to think of this is consciously putting yourself “in the other person’s shoes” each time you are assessing or interpreting your family member’s or friend’s health risk behaviour.

Try to focus on how your friend or family member **thinks* and feels*** about the health risk behaviour**. * See below for a definition of a thought and a feeling.**

Try to not let your own beliefs, values, or judgment of the health risk behaviour influence your understanding of the health risk behaviour from the family member’s or friend’s viewpoint. The next step should help ….

**Step 3. Identifying ‘cues’ as motivators for the health risk behaviour**

With (*insert health risk behaviour*), what kind of cues are you relying on to help you understand why your family member or friend does this? For example, is it usually a verbal cue or when your friend or family member has said something to you? Is there something else that triggers your friend or family member to engage in this health risk behaviour?

Here’s an example of *what you’d say in your own mind as you consider cues as viewed from the family member’s or friend’s perspective:*

 “I smoke because it provides me with stress relief. It gives me time to relax and get away for a moment. It allows consistent time to meet with specific friends.” 

Understanding cues allows you to better suggest meaningful changes to help the person overcome unhealthy behaviors.

This step should help you to think about how you recognize the family member’s or friend’s motivating factors to engage in the health risk behaviour from that individual’s viewpoint.

**Step 4: Validating your inferences about how the family member or friend views the health risk behaviour.**

You should try to validate your thoughts with him or her as you practice.

You can ask them if what you are noticing is close to what they are feeling. This step in validation is another part of learning perspective-taking.

I would encourage you to keep some notes about your thoughts and feelings when you attempt to picture how your friend or family member experiences the health risk behaviour over the next week.

**Remember: Your goal is to practice helping the family member or friend TALK through the ‘why’ of the health risk behaviour, ‘how’ the person is feeling, and ‘plans’ the person might have for change. You are NOT to elicit a change in the family member’s or friend’s thoughts, feelings, or motives to seek change.**

**POSSIBLE QUESTIONS FOR PERSPECTIVE-TAKING SCRIPT (STUDENT-CAREGIVER)**

**Developed by: Ms. Wilma Schroeder**

1.**Establishing Rapport:**

Use good manners, introduce yourself and make the person comfortable.

2**. Initiating the Discussion:** What health risk behaviour did you want to discuss today?

**3. Exploring the Health Risk:** Aim to have a conversation, rather reading off a questionnaire.

*Here are some questions as suggestions for you:*

What is most important thing about the health risk behaviour for you?

What do you like most about it?

What is the hardest thing about it?

How do you feel you feel when someone tells you to stop or cut down on the (identify the health risk behaviour)?

What would happen if you changed the health risk behaviour?

*Other open-ended questions:*

Tell me more about that

So what you are saying is… (paraphrase or re-state)

You feel… (paraphrase or restate)

That must be … (state a feeling/ e.g., said, frustrating, etc.)

Remember your non-verbal communication!

**4.Terminating the Interaction**

Our time is almost up. I’d like to summarize what we discussed. (briefly recap the interaction’s key points)

Did you have any other questions or anything to add?

Thank you for talking with me today.

**DEFNITION FOR A ‘THOUGHT’ AND A ‘FEELING’**

***Thought:*** *an idea, plan, opinion, picture, etc. that is formed in your mind: something that you think of (http://www.merriam-webster.com/dictionary/thought)*

***E.g., I was thinking that I needed to explain myself better; I was thinking I was interested in what she had to say.***

***Feeling:*** *an emotional state or reaction (http://www.merriam-webster.com/dictionary/feeling)*

***E.g., I was feeling tension because there was no conversation between us; I was feeling embarrassed because it seems like I should know better.***

**Note: these definitions were also provided during the video-tagging exercise**

## Measures

In the following, we provide additional details about the antecedents, interpersonal outcomes, and intrapersonal outcome measures that we used from the adapted Davis’s (1994) organizational model on empathy.

**Antecedents**

The preliminary questionnaire started with demographics capturing age, gender, ethnicity, extent of communication training with patients, communication training about health behavior, and participants’ health-risk behavior and desire to change.

Next, participants completed a modified Preventative Medicine Attitudes and Activities Questionnaire (Yeazel et al., 2006). This measured participants’ perceptions of preventative counseling effectiveness. We used three sub-scales: Behavior Change Effectiveness, Lifestyle Counseling Effectiveness, and Importance. The 7-item Behavior Change Effectiveness subscale measured perceptions of effectiveness at changing patient’s behaviors on a 5-pt scale (1 = *very effective*, 4 = *minimally effective* and 5 = *will not counsel*; α = .83; example item: “How effective will you be in changing your patients’ behavior with respect to: Exercise”). This subscale was adapted by reducing the number of health-risk behaviors from 15 to 7 through focusing on behaviors specific to the present study, and by changing the end anchor point from “do not counsel” to “will not counsel” given that participants were students with limited experience. The 12-item Lifestyle Counseling Effectiveness subscale measured attitudes towards counseling and health education on a 5-pt scale (1 = *strongly agree*, 5 = *strongly disagree*; α = .51; example item: “It is difficult for patients to make lifestyle changes”). This subscale was adapted by referring to providers instead of physicians; small wording changes; and by implementing changes made by Sannes (2011). Specifically, adding the questions “It is difficult for patients to make lifestyle changes”, “It is difficult to understand why patients can’t meet the goals they have set with you”, “I feel I have had a sufficient amount of training in motivational interviewing”, “I am able to identify the stage of change the patient is in to start applying motivational interviewing”, “Patients prefer being told what to do over helping to come up with a plan themselves”, “It is difficult for patients to adhere to their commitment to making lifestyle changes, despite being motivated at the start”, “Doing lifestyle counseling using motivational interviewing takes longer than traditional methods”; and removing questions relating to drug use, sex, AIDS, and sexual orientation. Items phrased negatively (e.g., “It is difficult for patients to make lifestyle changes”) were reverse-coded before combining. The 14-item Importance subscale was adapted to measure perceptions of the importance of asking about health behaviors and counseling patients on a 4-pt scale (1 = *very important, 4 = not very important*; α_ask_ = .82; α_counsel_ = .83; example items: “In general, how important is it for healthcare providers [e.g., general practitioner, counselor, etc] to ASK patients about the following: Exercise”, “In general, how important is it for healthcare providers [e.g., general practitioner, counselor, etc] to COUNSEL patients about the following: Exercise”). This subscale was adapted by referring to providers instead of physicians; by reducing the number of health-risk behaviors from 15 to 7; and by questioning about both asking and counseling patients. Composite scores were created by averaging items.

Next, participants completed the Positive and Negative Affect Scale (Watson et al., 1988). This measured participants’ positive and negative affect on a 20-item 5-pt scale (1 = *very slightly* *or not at all*, 5 = *extremely*; α_positive affect_ = .90; α_negative affect_ = .75; example items: “Indicate to what extent you feel this way right now, that is in the present moment: Interested”, “Indicate to what extent you feel this way right now, that is in the present moment: Distressed”). Composite scores were created by summing items.

**Interpersonal outcomes**

As an interpersonal outcome, participants completed the modified Consultation and Relational Empathy (CARE; Fitzgerald et al., 2014; Mercer et al., 2004). This measured their expected clinical empathy for the upcoming dialogue on a 9-item 10-pt scale (1 = *poor*, 5 = *excellent,* 6 = *does not apply*; α = .91; example item: “Please rate how you feel the client actor will perceive you to be at making him or her feel at ease?”). Since participants were not supposed to try to change client behavior during the dialogue, the question “How you feel the client will perceive you to be at making a plan of action with him or her” (Fitzgerald et al., 2014; Mercer et al., 2004) was removed for this study to assure participants that they were not to make any plans. Participants completed the CARE measure again in the post-dialogue questionnaire, this time reflecting on their empathy during the dialogue (α = .87). Composite scores were created by summing items.

The client-actor completed two measures after the dialogue. They completed the 1-item 10-pt scale Readiness to Change Ruler to determine whether their readiness to change their behavior was altered due to the conversation with the participant (1 = *not prepared to change*, 10 = *already changing*; Zimmerman et al., 2000). They also completed the 10-item CARE to measure their perceptions of the participant’s clinical empathy during the dialogue (α = .92; example item: “How good was the Practitioner at really listening?”). All questions were asked including “How good was the Practitioner at making a plan of action with you” (Mercer et al., 2004) in the event that the student made a plan of action with the client despite instructions.

**Intrapersonal outcome**

To measure perceptual understanding, a trained coder compared the client-actor’s thoughts and feelings to what the participant thought the client-actor was thinking and feeling (e.g., Ickes, 2001) as derived through the tagging exercise. As client-actors had multiple thoughts and feelings throughout the dialogue, the accuracy of each individual thought/feeling was scored a 0 (essentially different content), 1 (similar, but not the same), or 2 (essentially the same content). The trained coder considered whether there was a match in thought or feeling, tone, and referenced situation. Total percentage scores were calculated by summing the score for each thought/feeling and dividing by the maximum points the participant could have obtained.

## Client-actor script

**Kyle Berman ‑ (full time student w/ part time job struggling with anxiety)**

**Instructions for Student Interviewers (participants)**

You are about to meet with Mr. Kyle Berman, who is a 23-year-old full time university student, who also works part time. He struggles with anxiety, which he says effects his physical and mental wellbeing, and reduces his productivity. His doctor has recommended physical activity as a way to improve his mental and physical health, but he has struggled (particularly in the winter months) to do so. He credits his anxiety and workload as reasons for his dormancy. You are about to meet with Kyle at the recommendation of his doctor.

**Presenting Situation (for the Actor)**

You are a full-time student with a part time job who is struggling with anxiety. When the student asks you how you are doing, you say that you “feel drained all the time”. “*No matter what is going on, I seem to feel overwhelmed. I end up just sitting and watching Netflix instead of getting anything productive done. I procrastinate until the last second, which only makes me more anxious, and I end up calling in sick to work because I’m so stressed out. My doctor has told me that being more physically active would help with my anxiety, but I don’t have enough mental or physical energy to get started.”* If asked how long you’ve been feeling “drained”, you reply that its become more of a problem since you started University, and that you’re worried about how your physical health is affecting your mental state and your relationships.

**Background to the Problem**

For many years, you struggled with your anxiety, but just accepted it as “how things are”. For a while you could cope, but around the start of University, you realized that something was wrong. The job at a book store, which you normally enjoyed, became extremely stressful. You found yourself calling in sick because you couldn’t get out of bed in the morning. Then, instead of spending the free time getting classwork done, you would just lay in bed all day and watch Netflix. It was also affecting your relationships. Your long-term girlfriend broke up with you, and you hardly made time for your friends anymore. Last summer, you finally spoke to your doctor about it, and he recommended increasing your physical activity, which at the time was at almost zero. He said that regular physical activity was the number one way to naturally improve anxiety. With school done for the summer, and work being your only responsibility, you started going for long (2 hour+ walks multiple times per week) and hikes when you could manage it. You began feeling better physically and mentally. You had more energy and became more productive. You started to enjoy going to work again, made more time for friends, and even had extra time to pursue your passion for writing. Then the summer came to an end, classes (and the readings and essays) started taking up most of your time, and the weather got colder and colder until it wasn’t comfortable to go on your long walks even if you found the time to do so. Slowly but surely, your anxiety started creeping in again. All the old problems returned, seemingly with a vengeance. Your doctor recommended joining a gym, but the thought of exercising in front of other people only increased your anxiety. You want to feel better again, but don’t know how to get there given all the current factors.

**Your Symptoms**

If asked specifically how the situation is affecting you, you've noticed a number of things:

1. You are finding it increasingly difficult to stay motivated. You know being more active would decrease your anxiety and improve all aspects of your life (school, work, relationships), but the winter weather has removed your ability to do the types of activity you enjoy
2. Because you feel anxious, you procrastinate on your assignments, which only increases your anxiety. Instead of being productive, you lay in bed and watch TV. You also eat a lot of junk because it gives you temporary relief.
3. Your social anxiety makes it difficult to maintain relationships with friends and family, and also makes it difficult to go to a public gym because you don’t like the feeling of other people watching and judging you.

**Past Health**

You have no ongoing health problems other than your anxiety. You are not currently on medication for it, as you would like to attempt to improve things naturally first.

**Social Situation**

You live alone in a one-bedroom apartment. Your parents are supportive emotionally and financially, but you feel bad bugging them for help. You see them every few weeks, at their request. You rarely reach out yourself. You have an older sister who lives with her husband and two-year-old daughter. You love spending time with your niece when you have a chance. Usually your parents will offer to take you there when they’re going. You work with people you like, and have friends in your classes, but you don’t put much effort into spending time with them outside of school and work. Even if you make plans, you often cancel because your anxiety is bad.

You had a long-term relationship with your high school sweetheart, but she ended things a little over a year ago. You’re still sad about this. You’ve been on a few dates since, but you don’t feel ready to move on.

**Reasons for Wanting to Receive Physical Activity Counselling**

1. Feeling like your friends are no longer in frequent contact with you anymore because you don’t reach out to them very often, and when you have plans with them, you often cancel.
2. Last week you had your yearly evaluation at work, and your manager pointed out how many days you’d called in that year. She made it clear that it was something that you would have to improve.
3. You got an essay back recently with a grade of C+. This is well below what you know you’re capable of.
4. Because of the work you did last summer, you know how you’re capable of feeling when you are more physically active. You are hopeful that you might hear of some solutions that will work for you that you haven’t thought of.

**Affect and Response to Student**

You are nervous in the beginning. You’ve spoken a bit to your doctor about your issues, but you’re not used to opening up about your problems, especially to strangers.

Your feelings are complex. You feel guilty about letting other people down because of your anxiety. You’re angry that you can’t seem to take better care of yourself. You know being more physically active helps your mental and physical states, but you can’t seem to get yourself to do it. Getting stressed about not doing it only makes you spend another day in bed.

You want to blame the situation and make excuses. *“My brain is stopping me from being physically active.” “The weather makes it impossible to do the types of activity I like.” “Working out sucks and hurts.” “School and work don’t give me any time to be active.” “Aren’t I allowed to just relax?”*

It is the student’s nurse’s task to explore these complex feelings that are tied to your lack of physical exercise and increased anxiety, not to problem solve for you. If solutions are suggested prematurely, you will likely feel that the student doesn't understand your position and likely will resist the solutions. It is not possible to come up with an “instant-solve” solution to your problem that will fix everything at once.

**However, we need to reward a good student interviewing performance.** This means if you perceive the student asking open-ended questions and seeking to be helpful in an appropriate manner you should provide information more readily and at least agree to consider some of the options they suggest or try to have you come up with. You **do want help** - you've decided to talk about the problem and so should be amenable to some ideas.

## Video-Tagging Exercise – Single User Rating Sheet

**Instructions:**

**Target Partner**: The client-actor is assigned to watch their recorded dialogue. The client-actor is TO stop the video at 3 to 5 instances in the video where they experienced a thought or feeling. Using the below forced choice options for Situation, Thought or Feeling, and Tone; the time where they ‘tagged’ or annotated the video will be captured in the app.

**Perceiver Partner**: They will watch the video after the target partner watched the video. The perceiver will stop the video at each time point the target reported they stopped the video.

**Scoring:** The Rating (0 to 2) column is explained at the bottom of this table on how to provide a score from 0 (no agreement across 3 dimensions) to 1 (somewhat similar) to 2 (perfect agreement across 3 dimensions). See the formulae for calculating overall perceptual accuracy below.

| **Instance #**  **and Time Stamp** | **Sentence Describing the Situation** | **Thought or Feeling** | **Tone** | **Rating (0 to 2)** | **Rater Comments** |
| --- | --- | --- | --- | --- | --- |
| #1  ______mins | ___ self  ___ dialogue partner  ___ other person or persons  ___ current context of the dialogue  ___ other event or circumstances | __ Thought  __ Feeling | __ Positive  __ Negative |  |  |
| #2  ______mins | ___ self  ___ dialogue partner  ___ other person or persons  ___ current context of the dialogue  ___ other event or circumstances | __ Thought  __ Feeling | __ Positive  __ Negative |  |  |
| #3  ______mins | ___ self  ___ dialogue partner  ___ other person or persons  ___ current context of the dialogue  ___ other event or circumstances | __ Thought  __ Feeling | __ Positive  __ Negative |  |  |

**Key for Similarity Ratings-** 0 = (No agreement on situation, thought/feeling, or tone)

0 = (No agreement on situation or thought/feeling; agreement on tone)

0 = (No agreement on situation or tone; agreement on thought/feeling)

0 = (No agreement on situation; agreement on thought/feeling and tone)

1 = (Agreement on situation; no agreement on thought/feeling or tone)

1 = (Agreement on situation and thought/feeling; no agreement on tone)

1 = (Agreement on situation and tone; no agreement on thought/feeling)

2 = (Agreement on situation, thought/feeling, and tone)

**Hand Calculate the Overall Perceptual Accuracy Score (across all instances) (Ickes, 1993; 2001)**

(Sum of perceiver’s ratings) ÷ (Total # of Tags made by perceptual target × 2)] × 100% = Empathic Accuracy Measurement

**Supplemental table 1**. Participant and client-actor descriptive statistics by experimental condition.

| **Measure (Possible Range)** | **Full**  **Intervention** | | **Partial**  **Intervention** | |
| --- | --- | --- | --- | --- |
|  | **Mean (SD)** | **Range** | **Mean (SD)** | **Range** |
| Participant |  |  |  |  |
| PMAAQ Behavior Change Effectiveness (1 – 5) | 2.69 (0.63) | 1.29 – 4.14 | 2.69 (0.65) | 1.29 – 4.14 |
| PMAAQ Lifestyle Counseling Effectiveness (1 – 5) | 3.14 (0.38) | 2.33 – 4.08 | 3.04 (0.42) | 2.08 – 4.00 |
| PMAAQ Importance Ask (1 – 4) | 1.38 (0.41) | 1.00 – 2.71 | 1.42 (0.42) | 1 – 3 |
| PMAAQ Importance Counsel (1 – 4) | 1.40 (0.43) | 1 – 3 | 1.35 (0.39) | 1 – 2.86 |
| Positive Affect (10 – 50)* | 25.91 (7.30) | 12 – 43 | 26.71 (6.88) | 11 – 42 |
| Negative Affect (10 – 50) | 15.73 (4.33) | 10 – 32 | 15.42 (3.74) | 10 – 26 |
| Pre-Interview CARE (9 – 54)* | 28.78 (5.78) | 17 – 44 | 28.87 (5.44) | 9 – 41 |
| Post-Interview CARE (9 – 54)* | 26.71 (6.05) | 13 – 40 | 27.23 (5.13) | 18 – 41 |
| Perceptual Understanding (0 – 100)* | 50.81 (17.80) | 0.00 – 85.71 | 51.16 (18.68) | 5.88 – 81.82 |
| Client-actor |  |  |  |  |
| CARE (10 – 60)* | 26.28 (6.15) | 12 – 39 | 26.19 (6.39) | 14 – 44 |
| Readiness to Change (0 – 10)* | 5.59 (0.82) | 3.0 – 8.5 | 5.63 (0.90) | 2.5 – 8.0 |

*Note*. PMAAQ = Preventative Medicine Attitudes and Activities Questionnaire. CARE = Consultation and Relational Empathy. *Higher values are better

# References

Davis, M. H. (1994). *Empathy: A social psychological approach*. Madison, WI: Brown and Benchmark.

Fitzgerald, N., Heywood, S., Bikker, A., & Mercer, S. (2014). Enhancing empathy in healthcare: Mixed-method evaluation of a pilot project implementing the CARE Approach in primary and community care settings in Scotland. *Journal of Compassionate Health Care, 1*(1): 6. https://doi.org/10.1186/s40639-014-0006-8

Ickes, W. (2001). Measuring empathic accuracy. In, J. A. Hall & F. J. Bernieri (Eds.), *Interpersonal sensitivity: Theory and measurement*, pp. 219-241. New Jersey: Lawrence Erlbaum Associates.

Mercer, S. W., Maxwell, M., Heaney, D., Watt, G. C. (2004). The consultation and relational empathy (CARE) measure: development and preliminary validation and reliability of an empathy-based consultation process measure. *Family Practice. 21*(6), 699–705. https://doi.org/10.1093/fampra/cmh621

Sannes, H.J. (2011). Barriers to using motivational interviewing for lifestyle counseling. [Unpublished thesis, Minnesota State University). <https://cornerstone.lib.mnsu.edu/cgi/viewcontent.cgi?article=1210&context=etds>

Watson, D., Clark, L., & Tellegen, A. (1988). Development and Validation of Brief Measures of Positive and Negative Affect: The PANAS Scales. *Journal of Personality and Social Psychology, 54*(6), 1063–1070. https://doi.org/10.1037/00223514.54.6.1063

Yeazel, M., Lindstrom Bremer, K., & Center, B. (2006). A validated tool for gaining insight into clinicians’ preventive medicine behaviors and beliefs: The preventive medicine attitudes and activities questionnaire (PMAAQ). *Preventive Medicine, 43*(2), 86–91. https://doi.org/10.1016/j.ypmed.2006.03.021

Zimmerman, G., Olsen, C., & Bosworth, M. (2000). A “stages of change” approach to helping patients change behavior. *American Family Physician, 61*(5), 1409–1416. https://www.aafp.org/afp/2000/0301/p1409.html
